# The small GTPase ARL2 is required for cytokinesis in *Trypanosoma brucei*

**DOI:** 10.1016/j.molbiopara.2010.05.016

**Published:** 2010-10

**Authors:** Helen P. Price, Adam Peltan, Meg Stark, Deborah F. Smith

**Affiliations:** a Centre for Immunology and Infection, Department of Biology/Hull York Medical School, University of York, York YO10 5YW, UK; b Technology Facility, Department of Biology, University of York, Heslington, York YO10 5YW, UK

**Keywords:** ANT-1, adenine nucleotide transporter 1, Arf, ADP-ribosylation factor, Arl, ADP-ribosylation factor-like, BART, ARF-like 2-binding protein, BSF, bloodstream form, dsRNA, double-stranded RNA, ELMO, Engulfment and Cell Motility, ELMOD, Engulfment and Cell Motility Domain, ER, endoplasmic reticulum, FAZ, flagellum attachment zone, GAP, GTPase activating protein, HRG4, human retinal gene 4, NMT, myristoyl-CoA:protein *N*-myristoyltransferase, PP2A, protein phosphatase 2A, RNAi, RNA interference, RP2, retinitis pigmentosa 2, *Trypanosoma brucei*, Arl2, Cytokinesis, Tubulin acetylation

## Abstract

The Arf-like (Arl) small GTPases have a diverse range of functions in the eukaryotic cell. Metazoan Arl2 acts as a regulator of microtubule biogenesis, binding to the tubulin-specific chaperone cofactor D. Arl2 also has a mitochondrial function through its interactions with BART and ANT-1, the only member of the Ras superfamily to be found in this organelle to date. In the present study, we describe characterization of the Arl2 orthologue in the protozoan parasite *Trypanosoma brucei*. Modulation of TbARL2 expression in bloodstream form parasites by RNA interference (RNAi) causes inhibition of cleavage furrow formation, resulting in a severe defect in cytokinesis and the accumulation of multinucleated cells. RNAi of TbARL2 also results in loss of acetylated α-tubulin but not of total α-tubulin from cellular microtubules. While overexpression of TbARL2^myc^ also leads to a defect in cytokinesis, an excess of untagged protein has no effect on cell division, demonstrating the importance of the extreme C-terminus in correct function. TbARL2 overexpressing cells (either myc-tagged or untagged) have an increase in acetylated α-tubulin. Our data indicate that Arl2 has a fundamentally conserved role in trypanosome microtubule biogenesis that correlates with α-tubulin acetylation.

## Graphical abstract

The *Trypanosoma brucei* orthologue of Arl2 is essential for viability in bloodstream form parasites. RNA interference causes inhibition of cleavage furrow formation and loss of acetylated α-tubulin.

## 1 Introduction

The regulation of microtubule dynamics is critical for numerous cellular processes, including mitosis, vesicular transport and motility [1]. Microtubule formation involves the polymerisation of heterodimers composed of GTP-bound α- and β-tubulin to form protofilaments which assemble into cross-linked helical bundles. The energetic stability of a microtubule is decreased by hydrolysis of β-tubulin-associated GTP at the growing (+) end [2]. GTP hydrolysis is promoted by the tubulin-specific chaperones, cofactors C, D and E, which form a supercomplex with the tubulin αβ heterodimer and act as GTPase activating proteins (GAPs) [3]. Overexpression of cofactors D or E in cultured cells accelerate the rate of GTP hydrolysis by β-tubulin, resulting in microtubule instability and depolymerisation [4].

The ADP-ribosylation factor (ARF) family of small GTPases are a highly conserved group of *N*-myristoylated proteins associated primarily with roles in vesicle budding and membrane trafficking [5]. Two divergent members of this family, ADP-ribosylation factor-like 2 and 3 (Arl2 and Arl3), differ from other Arf/Arl proteins in that they lack an *N*-myristoylation site and both have been implicated in the regulation of microtubule biogenesis [6]. *Arl2* (but not *Arl3*) has been identified in genetic screens for regulating factors of microtubules in several model organisms [7–9]. Arl2 binds to cofactor D, inhibiting its GAP function in the tubulin-cofactor supercomplex and so inhibiting GTP hydrolysis by β-tubulin during the final stage of αβ tubulin heterodimer production. Approximately 90% of Arl2 protein in bovine brain exists as part of a 300 kDa complex, which also includes cofactor D and protein phosphatase 2A (PP2A) [10]. Analysis using nucleotide-locked mutants indicated that only the GDP-bound form of Arl2 was able to bind to cofactor D [4] but recent studies suggest that the interaction may not be nucleotide-dependent [11]. Modulating the expression level of Arl2 is sufficient to alter the protein level of its effector PP2Ac in breast cancer cells, with a corresponding modification of the phosphorylation status and microtubule association of downstream targets of the phosphatase, including the tumour suppressor p53 [12,13]. In addition to a regulatory role in microtubule dynamics, the Arl2/cofactor D/PP2A complex has been implicated in controlling assembly–disassembly of the apical junctional complex between epithelial cells [14].

Arl2 has a secondary role in the mitochondrion, the only member of the Ras superfamily known to function in this organelle to date [10]. GTP-bound Arl2 participates in a mitochondrial complex with Binder of ARL2 (BART) [15] and adenine nucleotide transporter 1 (ANT-1) [16]. ANT-1 has a key role in oxidative phosphorylation, regulating cytoplasmic ATP levels by exchanging cytoplasmic ADP for mitochondrial ATP [17]. A recent study on rat cardiac myocytes revealed Arl2 to be the target for a specific microRNA, miR-15b, which decreases cellular ATP levels without affecting cell viability. Further, knockdown of *Arl2* in cardiomyocytes causes abnormal mitochondrial morphology. The interaction of Arl2 and ANT-1 is therefore believed to be critical for the regulation of ATP levels in cardiac tissue [18]. In addition to this role, Arl2 and BART are known to be essential for nuclear retention of the transcription factor STAT3 [19]. BART was the first Arl2 effector to be identified and has no GAP activity [15]. While its exact function has yet to be elucidated, BART can bind to ANT-1 and STAT3 both independently and as part of an Arl2-BART complex and so may be acting as a small adapter molecule in this context [16,19]. Arl2 GAP function is provided in the mitochondrion by at least two proteins, Engulfment and Cell Motility Domain 1 and 2 (ELMOD1 and ELMOD2), which demonstrate activity against Arl2, Arl3 and Arf1, despite lacking the canonical zinc finger Arf GAP motif [20]. ELMOD1 and ELMOD2 form part of a group of six human proteins which contain an Engulfment and Cell Motility (ELMO) domain (or DUF609), a conserved region of unknown function associated with proteins functioning in cell migration and the phagocytosis of apoptotic cells [20]. BART and the ELMOD proteins are not evident in early eukaryotes [20], indicating either significant divergence in these molecules or restriction of the mitochondrial function of Arl2 to metazoans.

The related GTPase Arl3 has the ability to bind all Arl2 effector proteins except for cofactor D [10] but the two proteins are functionally distinct. Binding specificity is achieved through the formation of a ternary complex between Arl3 and its specific interaction partners, human retinal gene 4 (HRG4/UNC119) and retinitis pigmentosa 2 (RP2), an Arl3-specific GAP with sequence identity and functional homology to cofactor C [11]. Arl3 is associated with primary cilia in human photoreceptor cells [21,22] and deletion of the *Arl3* gene in mice results in photoreceptor degeneration [23]. An orthologue of Arl3 has also been linked to flagellum development in the protozoan parasites *Leishmania donovani* and *Trypanosoma brucei* [24,25].

In the current study, we have investigated the functions of Arl2 in *T. brucei*, an organism which is dependent on a largely tubulin-based cytoskeleton [26]. We show that modulation of Arl2 levels by RNA interference (RNAi) inhibits the formation and ingression of cleavage furrows, resulting in a severe defect in cytokinesis. Overproduction of untagged TbARL2 (TbARL2^NoTag^) has no effect on cell growth and morphology but expression of C-terminal myc-tagged protein (TbARL2^myc^) inhibits cleavage ingression, leading to a defect in cytokinesis. We also report that altering the level of TbARL2 expression is sufficient to modulate the amount of acetylated tubulin detected in the cytoskeleton of this ancient eukaryote.

## 2 Materials and methods

### 2.1 Parasite culture

*T. brucei* bloodstream form (BSF) strain Lister 427 (Single Marker Bloodstream) was maintained as described [27]. This strain stably expresses a phage derived T7 RNA polymerase and tetracycline repressor.

### 2.2 DNA constructs

The plasmid vectors p2T7Ti [28] and pT7-^MYC-C^ (also called pM2cC) [29,30] were gifts from Doug LaCount (PULSe, Purdue University, West Lafayette, IN, USA) and David Horn and Sam Alsford (London School of Hygiene and Tropical Medicine, London, UK), respectively. The RNAi vector p2T7Ti supports expression of double-stranded RNA from two opposing tetracycline-inducible T7 promoters. Vector pT7-^MYC-C^ is used to overexpress the target gene with a C-terminal myc epitope tag under the control of a tetracycline-inducible T7 promoter. Both vectors contain flanking regions for integration into the transcriptionally silent rDNA spacer regions of the *T. brucei* genome. All primer sequences are provided in Supplementary Table 1. A non-conserved region of the *T. brucei ARL2* gene (*Tb10.70.3000*) corresponding to residues 1–247 of the open reading frame was identified using the program RNAit [31]. The fragment was amplified from *T. brucei* genomic DNA using primers A2-RNAi-F and A2-RNAi-R and ligated into the plasmid p2T7Ti [32] to produce the construct p2T7ARL2. The *TbARL2* ORF was amplified from genomic DNA using primers A2-myc-F and A2-myc-R and ligated into plasmid pT7-^MYC-C^ to produce the construct pARL2^MYC^. T31N and Q70L mutations were introduced using the GeneTailor Site-Directed Mutagenesis System (Invitrogen) and primers A2-T31N-F, A2-T31N-R, A2-Q70L-F and A2-Q70L-R. A construct for overexpression of non-tagged TbARL2 (pARL2^NoTag^) was produced by the introduction of a stop codon upstream of the C-terminal myc epitope tag in pARL2^MYC^ using the primers A2-Untagged-F and A2-Untagged-R (Supplementary Table 1).

### 2.3 Parasite transfection

Mid-log BSF cells were transfected by electroporation with *Not*I-digested p2T7ARL2, pARL2^MYC^ or pARL2^NoTag^ as described previously [33]. Stable transformants were selected by growth in 2.5 μg/ml phleomycin (RNAi) or 10 μg/ml hygromycin (overexpression). Expression of dsRNA or ARL2 protein was induced by the addition of tetracycline (1 μg/ml). Cells were counted using a Beckman Coulter counter and cumulative growth plotted as described [34]. Cell viability was tested at 0–40 h post-induction using a Live/Dead Viability/Cytotoxicity kit for mammalian cells (Invitrogen), according to the manufacturer's instructions [35,36]. Subsequent flow cytometry analysis (10,000 cells per sample) was performed on a Dako CyAn with FL1 and FL3 detectors and results analysed with Summit v4.1 software. Immunoblotting was performed on total lysates from parasites grown in the absence or presence of tetracycline for 16 h, as described previously [30].

### 2.4 Quantitative PCR (qPCR)

Absolute quantitation by qPCR was used to determine changes in ARL2-specific transcript following tetracycline induction, relative to a constitutively expressed control, either α-tubulin or myristoyl-CoA:protein *N*-myristoyltransferase (NMT). Total RNA was extracted from parasites using Trizol reagent (Invitrogen) as described by the manufacturer. Traces of genomic DNA were removed by treatment with DNase I, prior to reverse transcription using Omniscript RT (Qiagen) and Oligo-dT (Promega). The program Primer Express (Applied Biosystems) was used to design the following primers: A2-qPCR-F, A2-qPCR-R, α-Tub-qPCR-F, α-Tub-qPCR-R, NMT-qPCR-F and NMT-qPCR-R (Supplementary Table 1). Quantitative PCR reactions were performed using SYBR Green Mastermix (Applied Biosystems) on an ABI 7000 Sequence Detection System (Applied Biosystems) and results analysed with Sequence Detection Software v1.2.3 (Applied Biosystems).

### 2.5 Microscopy and flow cytometry

For cell cycle analysis, parasites were stained with DAPI (1.5 μg/ml) and the number of kinetoplasts and nuclei were counted in at least 250 cells per sample, using a Nikon Eclipse E600 microscope with a Plan-Fluor 100×/1.30 objective lens. Propidium iodide staining of cells was performed as described previously [37], prior to analysis by flow cytometry (50,000 cells per sample) on a Dako CyAn using the FL3 detector.

Transmission electron microscopy was performed as described previously [36]. For scanning electron microscopy, cells were fixed in 1% gluteraldehyde for 1 h then washed twice for 30 min in 100 mM phosphate buffer. All steps were performed in microcentrifuge tubes, briefly centrifuged and resuspended between each step. Cells were then dehydrated by suspending in an ethanol series of 50%, 70%, 90%, 100% for 30 min at each step, before addition of hexamethyl disilazane (HMDS) for 30 min. Cells were then air dried overnight. The pellet was vortexed, mounted on aluminium SEM stubs, coated with a thin layer of gold/palladium and visualised on a JEOL JSM-649OLV scanning electron microscope at 8 kV, spot size 35.

For tubulin analysis, indirect immunofluorescence assays were performed on fixed parasites in suspension. The mouse monoclonal antibody TAT1 (*T. brucei* α-tubulin, dilution of 1:200) was a gift from Keith Gull (Sir William Dunn School of Pathology, University of Oxford, UK). Rat anti-tyrosinated α-tubulin clone YL1/2 (AbD Serotec) and mouse anti-acetylated α-tubulin clone 6-11B-1 (Sigma) were both used at a dilution of 1:250. Primary antibodies were detected using Alexa Fluor 488-conjugated secondary antibodies (Invitrogen). Briefly, cells (1 × 10^7^ per sample) were fixed in 4% paraformaldehyde/PBS on ice for 45 min, then washed in PBS. Samples were incubated in 0.2% Triton X-100/PBS for 10 min at RT, then in blocking buffer (10% FCS/PBS) on ice for 20 min. Cells were probed with primary antibody diluted in blocking buffer on ice for 20 min, washed 3× in PBS, then incubated in secondary antibody diluted in blocking buffer for a further 20 min on ice. Following washing in PBS, parasite samples were divided into two and analysed by confocal microscopy and flow cytometry. Samples were visualised using a Zeiss LSM 510 meta with a Plan-Apochromat 63×/1.4 oil DIC I objective lens. Images were acquired using LSM 510 version 3.2 software, using identical settings for all samples stained with each antibody. Flow cytometry analysis (50,000 cells per sample) was performed on a Dako CyAn with FL1 detector and results analysed with Summit v4.1 software.

### 2.6 Subcellular fractionation

Cytoskeleton and flagellar fractions were prepared by subjecting cells to detergent/NaCl extraction as described previously [38]. Briefly, parasites stably transfected with pARL2^MYC^ were grown in the presence or absence of tetracycline for 16 h, centrifuged at 800 × *g* for 10 min at 20 °C, then washed in PBS. Following centrifugation as above, cells were resuspended in PEME (100 mM PIPES, 2 mM EGTA, 0.1 mM EDTA and 1 mM MgSO4, pH 6.9) containing 1% Nonidet P40, 1× Complete protease inhibitor cocktail (Roche), 7.5 μM Pepstatin A and 5 μM E-64d. Parasites were incubated on ice for 10 min, then centrifuged at 15,000 × *g* for 15 min at 4 °C. Pellets were either washed twice in PEME and resuspended in Laemmli buffer (cytoskeleton fraction) or further extracted in PEME containing 1 M NaCl, 200 μg/ml DNaseI, 50 μg/ml RNaseA and protease inhibitors as above. Samples were incubated on ice for 10 min, centrifuged as above, salt extraction repeated once, then pellets washed twice in PEME. Pellets were resuspended in Laemmli buffer (flagellar fraction). Total cell lysates, cytoskeletal and flagellar extracts from the equivalent of 1 × 10^7^ cells per sample were analysed by immunoblotting and probed with the following mouse monoclonal antibodies: anti-myc (Invitrogen, 1:2000), anti-elongation factor-1α (EF-1α) clone CBP-KK1 (Millipore, 1:2000) and anti-PFR1/2 clone L13D6 (a gift from Keith Gull, Sir William Dunn School of Pathology, University of Oxford, UK, 1:500).

## 3 Results

### 3.1 Bioinformatics

BLAST searches were performed on the GeneDB genome databases of *T. brucei* and related kinetoplastid species to identity orthologues of Arl2 and known interacting partners of this protein. The *T. brucei* Arl2 orthologue TbARL2 shares 63% identity at the amino acid level with human Arl2 and contains many of the conserved residues seen in other species (Supplementary Fig. 1). With the exception of PP2A subunit BB′ɛ, putative orthologues of all known Arl2 effector proteins were identified in the kinetoplastid species (Supplementary Table 1). It is interesting to note that putative orthologues of the mitochondrial proteins BART and ELMOD2 were identified in the kinetoplastids by BLAST searches (Supplementary Fig. 2 and Supplementary Table 2) but could not be detected in other lower eukaryote species (*S. cerevisiae*, *S. pombe*, *P. falciparum* and *E. histolytica*).

The ELMOD orthologues share a high degree of evolutionary conservation, although unlike other proteins in this family, one of the *T. brucei* sequences is predicted to be *N*-myristoylated [39], a factor which may aid reversible binding to membranes. In contrast, the putative BART orthologues in kinetoplastids are significantly divergent from those found in higher eukaryotes. However, all identified BART proteins have a common predicted structure, containing six alpha helices (Supplementary Fig. 2) which correlates with the elucidated crystal structure of human BART [40,41]. Further studies will be required to determine the relationships between the putative orthologues of ELMOD2, BART and Arl2 and whether these molecules play a functional role in the kinetoplast mitochondrion as in higher eukaryotes.

### 3.2 TbARL2 is essential for cytokinesis

**Fig. 1:**
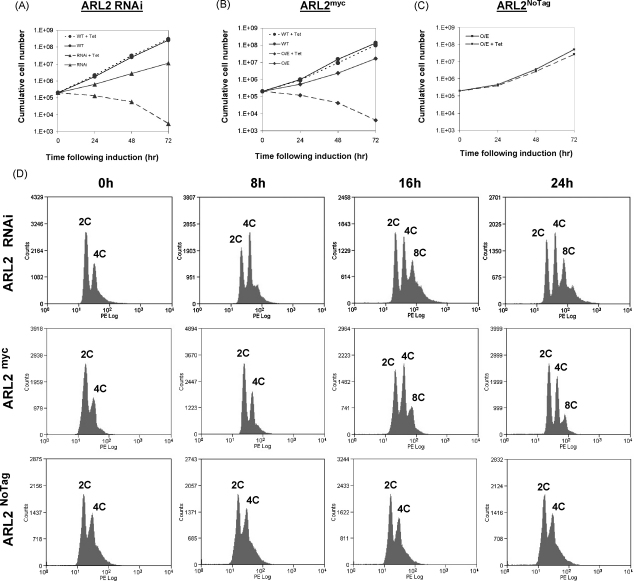
Effects of TbARL2 RNAi and overexpression on cell growth and division in *T. brucei* bloodstream form (BSF) parasites. (A–C) Cumulative growth of BSF parental line Lister 427 (WT) and transfected lines 427/p2T7ARL2 (ARL2 RNAi), 427/pARL2^myc^ (overexpression of C-terminal myc-tagged protein TbARL2^myc^) and 427/pARL2^NoTag^ (overexpression of untagged protein) respectively in the absence and presence of tetracycline, monitored over a 3-day time course. (D) Flow cytometry of propidium iodide stained cell lines as above, grown in the presence of tetracycline for 0–24 h. DNA content of each peak is shown.

We investigated the functions of TbARL2 in bloodstream form (BSF) cells by the tetracycline induction of RNA interference (RNAi) or overexpression of either a C-terminally myc-tagged form (TbARL2^myc^) or an untagged form of the protein (TbARL2^NoTag^). Both RNAi and overexpression of TbARL2^myc^ caused a rapid inhibition of cell division, with little or no increase in parasite numbers by 24 h post-induction (Fig. 1A and B). However, a two-colour fluorescent cell viability assay showed that, in both cases, over 90% of the parasites were still viable by 24 h post-induction, and approximately 80% viable by 40 h post-induction (data not shown), indicating a cytostatic rather than cytotoxic effect. In contrast, overexpression of TbARL2^NoTag^ had no effect on cell growth (Fig. 1C). This finding is in agreement with a recent paper describing the deleterious effects of epitope tagging on the functions of ARF family proteins including human Arl2 [42] and indicates that the extreme C-terminus of TbARL2 is important for correct function of the protein.

As an antibody was not available for detection of endogenous and untagged TbARL2, quantitative PCR was used as an indication of the efficiency of RNA knockdown and overexpression, with *N*-myristoyltransferase (NMT) as the constitutively expressed control (Supplementary Fig. 3A and B). Cells undergoing knockdown showed a decrease in Arl2-specific transcript to about 42% of the original level by 24 h post-induction (Supplementary Fig. 3A). In comparison, induction of overexpression caused a 4.6-fold increase in Arl2-specific transcript in TbARL2^myc^ expressing cells and a 14.4-fold in the TbARL2^NoTag^ line within this time (Supplementary Fig. 3B). Overexpression of TbARL2^myc^ (and of subsequent myc-tagged mutant proteins) was detected by immunoblotting using an anti-myc antibody (Supplementary Fig. 3C). We found that TbARL2^myc^ expressing cell lines were relatively unstable, losing the ability to inducibly express the tagged protein if cultured *in vitro* over an extended period of time (>6 weeks). Therefore, all experiments using these lines were performed using cells grown for less than 4 weeks in culture following transfection.

Both knockdown and expression of TbARL2^myc^ had a dramatic effect on cell cycle progression, as assessed by DNA content measurement by flow cytometry (Fig. 1D) and microscopy (Supplementary Fig. 3D and E) but no differences were observed for cells overproducing TbARL2^NoTag^ (Fig. 1D). As trypanosomes replicate, they undergo an asymmetric replication in which the kinetoplast (mitochondrial DNA) divides post S-phase prior to the nucleus. Cells therefore progress from a configuration of one kinetoplast and one nucleus (1K1N) to two kinetoplasts and one nucleus (2K1N). The nucleus divides at mitosis to produce cells with two kinetoplasts and two nuclei (2K2N) which then undergo cytokinesis to form two daughter cells (1K1N). Cell cycle progression was studied by flow cytometry analysis of propidium iodide stained parasites (Fig. 1D). This showed that the majority of uninduced cells had a DNA content of 2C (corresponding to a single diploid nucleus). Tetracycline induction of ARL2 RNAi resulted in an accumulation of cells with a DNA content of 4C (corresponding to two diploid nuclei) by 8 h (the period of one round of division), while no obvious effect was seen in cells expressing TbARL2^myc^ at this timepoint by flow cytometry. By 24 h post-induction, a significant proportion of both the RNAi and TbARL2^myc^ expressing cells had a DNA content of 4C, 8C or greater, indicating multiple nuclei (Fig. 1D). Accumulation of multinuclear cells in these samples was confirmed by microscopy (Supplementary Fig. 3D and E). Therefore, these cells had reached G2/M phase, were unable to proceed through cytokinesis but were capable of proceeding unchecked into the next G1 phase.

In order to determine if the effects of TbARL2^myc^ expression were affected by nucleotide configuration, mutant forms of the protein, T31N (GDP-locked) and Q70L (GTP-locked), were also inducibly expressed in *T. brucei*. Both of these led to a cessation in cell division (Supplementary Fig. 3F), with the T31N mutant protein causing the most severe phenotype of the two. Flow cytometry analysis of DNA content showed that expression of either of the mutant proteins resulted in the accumulation of abnormal cells with multiple nuclei by 16 h post-induction (Supplementary Fig. 3G). We were therefore unable to establish the relative importance of nucleotide binding state by this analysis.

**Fig. 2:**
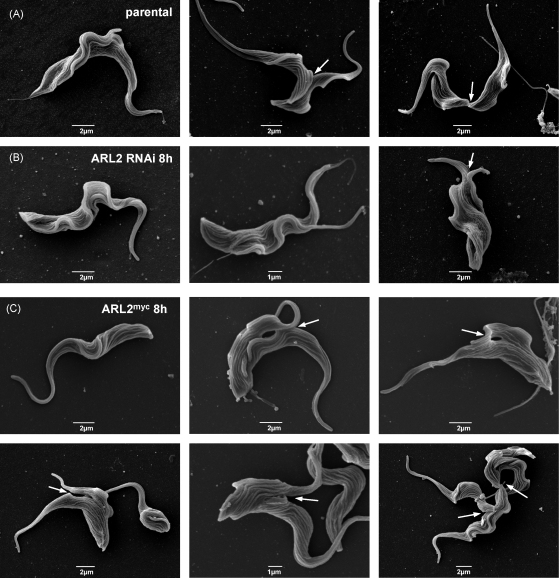
Scanning electron micrographs of *T. brucei* BSF parental line (A) and cell lines 427/p2T7ARL2 (B) and 427/pARL2^myc^ (C) grown in the presence of tetracycline for 8 h. Cytokinetic cleavage furrows are indicated by arrows.

The effects on cytokinesis were studied in detail by scanning electron microscopy in RNAi and TbARL2^myc^ expressing cells (Fig. 2). In the parental line, parasites could be observed spanning all stages of the cell cycle, including examples with ingressing cytokinetic cleavage furrows (Fig. 2A). At 8 h following the induction of TbARL2 RNAi, cleavage furrows were extremely difficult to detect, despite the analysis of several hundreds of cells. Further, the furrows which could be detected appeared to have attenuated ingression (Fig. 2B). These results correlate with DNA content analysis (Fig. 1D) which shows the accumulation of cells with two nuclei at 8 h, followed by the appearance of multinucleated parasites at later timepoints. In contrast, ingressing furrows were clearly detected in TbARL2^myc^ expressing cells at 8 h post-induction (Fig. 2C). These cells appear to have a failure in cytokinesis at a later stage (Fig. 2C) therefore TbARL2^myc^ is unlikely simply to be imposing a dominant-negative effect. These data together indicate that TbARL2 is required for correct furrow formation and ingression. In the absence of the protein, furrow formation is severely inhibited whereas in the presence of incorrectly functioning TbARL2^myc^ cleavage furrow ingression is initiated but not completed and the daughter cells fail to separate. The effects of inducing these two processes are different but both lead ultimately to cytokinesis failure and the production of multinucleated cells as a consequence of disturbing normal ARL2 function.

Defects in cytokinesis have been described in bloodstream form *T. brucei* following the knockdown of a range of genes involved in cell cycle regulation, flagellar morphogenesis and other processes [38,43–45]. These knockdowns may affect cytokinesis directly (e.g. MOB1, required for furrowing [43]) or indirectly (e.g. Centrin 1, causing defects in basal body and Golgi duplication [46]). The data we present here suggest that Arl2 has a direct effect on cleavage furrow formation, potentially due to disruption of microtubule dynamics.

Transmission electron microscopy was also used to visualise the effects of Arl2 RNAi on cell morphology. As expected, a large proportion of induced cells were observed to have multiple nuclei and flagella following the induction of Arl2 RNAi for 24 h (Supplementary Fig. 4B). However, no other dominant morphological changes were observed. Flagellar structural defects including internal flagella (Supplementary Fig. 4A and C) or microtubule disorder (Supplementary Fig. 4D) were observed in approximately 5% of cells by 24 h post-induction (approximately 200 cells scored per sample). The subpellicular microtubules were observed to be intact and ordered, as was the flagellum attachment zone (FAZ), a specialized structure connecting the flagellum with the cell body, which is made up of a dense filament and four ER-associated microtubules [47]. No gross morphological changes were observed in the Golgi apparatus (Supplementary Fig. 4D). Indirect immunofluorescence assays were also used to determine the effects of altering TbARL2 expression on subcellular compartments of the parasite. The endoplasmic reticulum, lysosome and early endosomes continued to replicate unchecked despite a defect in cytokinesis. Markers of these compartments (BiP, p67 and Rab5, respectively) became distributed in a widespread punctate pattern throughout cells, following induction of either RNAi or expression of TbARL2^myc^ (data not shown).

### 3.3 TbARL2 expression level influences tubulin acetylation

**Fig. 3:**
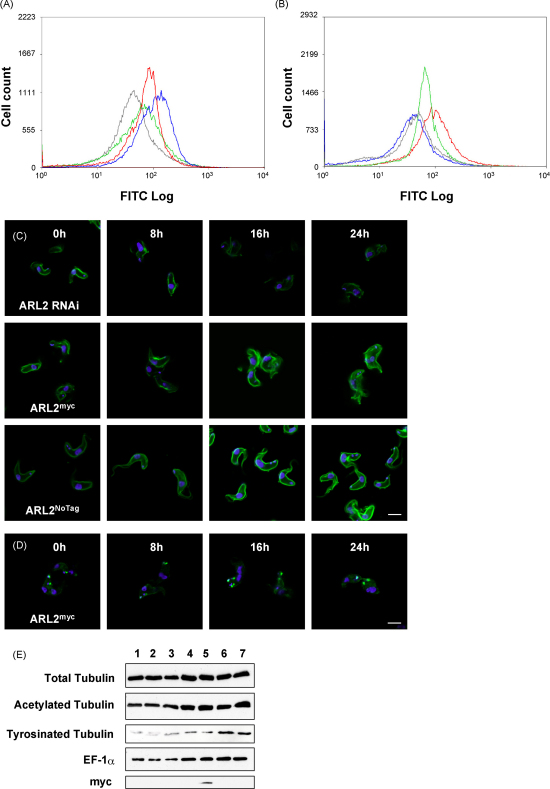
Effects of modulating TbARL2 expression on tubulin. (A and B) Flow cytometry of cells stained with antibodies against total α-tubulin (A) and acetylated α-tubulin (B) and detected using Alexa Fluor 488-conjugated goat-anti-mouse. Grey, parental line. Blue, 427/p2T7ARL2 (RNAi). Red, 427/pARL2^MYC^ and Green, 427/pARL2^NoTag^ (overexpression). All lines were grown in the presence of tetracycline for 16 h. (C and D) Immunofluorescence analysis of cell lines as above grown in the presence (+Tet) of tetracycline for 0–24 h. Cells were stained with antibodies against acetylated α-tubulin (C) and tyrosinated α-tubulin (D) and co-stained with DAPI. Bar, 5 μm. (E) Total lysates of cells grown in the absence or presence of tetracycline for 16 h were immunoblotted (lysate from 1 × 10^5^ cells/lane, except for blot probed with anti-myc in which 1 × 10^7^ cells were loaded per lane) and probed with antibodies against total α-tubulin (TAT1), acetylated α-tubulin, tyrosinated α-tubulin and myc epitope. Anti-EF-1α was used to monitor equal sample loading. Lane 1, Lister 427 parental line. 2 and 3, 427/p2T7ARL2 (RNAi). 4 and 5, 427/pARL2^MYC^. 6 and 7, 427/pARL2^NoTag^. Uninduced cell lysates are shown in lanes 1, 2, 4, 6. Lysates from cells grown in tetracycline for 16 h are shown in lanes 3, 5 and 7.

Modulation of TbARL2 levels by RNAi or overexpression of TbARL2^myc^ or TbARL2^NoTag^ resulted in a notable increase in the intensity of total α-tubulin by 16 h post-induction, as measured by flow cytometry (Fig. 3A). This may be due to an increase in the rate of tubulin polymerisation or a decrease in the rate of microtubule disassembly. Cells were also probed with the YL1/2 antibody which is specific for tyrosinated α-tubulin and therefore a marker for newly assembled microtubules, such as those associated with newly formed flagella in *T. brucei* [48], plus an aggregation of unpolymerised tubulin adjacent to the basal bodies [49]. Induction of both overexpression (Fig. 3D) and RNAi (data not shown) of TbARL2 caused an increase in the number of foci in each cell, a pattern consistent with unchecked duplication of basal bodies in the absence of cytokinesis in these parasites.

In contrast, the level of acetylated tubulin appears to be directly influenced by the level of TbARL2 expression, with an increase in the intensity of modified tubulin staining in detergent extracted cells following overexpression (TbARL2^myc^ or TbARL2^NoTag^) and a decrease following RNAi, as analysed by flow cytometry and confocal microscopy (Fig. 3B and C). A previous study reported that knockdown of human Arl3 led to an increase in acetylated tubulin [50] but Arl2 has not previously been linked to this post-translational modification. We also analysed total cellular tubulin by immunoblotting (Fig. 3E). The levels of total, acetylated and tyrosinated α-tubulin were measured by densitometry, relative to a constitutively expressed marker EF-1α. While the levels of total cellular α-tubulin were observed to be approximately the same in all lines, the relative amount of acetylated α-tubulin increased 2-fold in ARL2^NoTag^ overexpressing cells by 16 h post-induction compared to uninduced cells (Fig. 3E, lanes 6 and 7). Surprisingly, the largest observed differences were in the relative amount of cellular tyrosinated α-tubulin, which was 6-fold higher in the ARL2^NoTag^ cell line (both uninduced and induced) compared to the other analysed lines (Fig. 3E).

### 3.4 TbARL2^myc^ is detected in cytoskeletal fractions

Identifying the precise subcellular localization of TbARL2 will be key for elucidating its function in the trypanosome. However, no specific antibody is currently available for TbARL2 and a commercial polyclonal antibody against human ARL2 (ab71288, AbCam) recognises a number of additional proteins in total parasite lysates (data not shown). The subcellular localization of TbARL2 was therefore investigated in cells overexpressing TbARL2^myc^. Immunofluorescence revealed punctate staining throughout the parasite, which showed no significant co-localization with markers of the ER or endosomes (data not shown). Our analysis was partly hindered by the detrimental effects of overexpressing this protein on parasite morphology. Attempts to rectify this by decreasing tetracycline concentration or incubation time resulted only in expression of the tagged protein at barely detectable levels. This problem has been encountered previously for the related GTPase TbARF1, overexpression of which is highly toxic to the cell, even at very low levels [36].

**Fig. 4:**
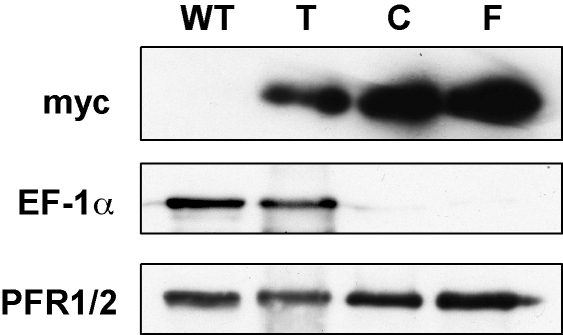
Subcellular fractionation of TbARL2^myc^. Prepared subcellular fractions from equivalent numbers of cells were separated by SDS-PAGE, transferred to PVDF membrane and the resulting immunoblot probed with mouse anti-myc, anti-EF-1α and anti-PFR1/2. Analysed samples were total cell lysate from parental line Lister 427 (WT) and subcellular fractions from cell line 427/pARL2^MYC^ grown in the presence of tetracycline for 16 h: total cell lysate (T), cytoskeletal fraction (C) and flagellar fraction (F).

Subcellular fractionation was therefore used as an additional approach to investigate intracellular localization. TbARL2^myc^ was retained following extraction with detergent and 1 M NaCl (Fig. 4B), indicating that the majority of the protein is associated with the cytoskeleton, specifically in the highly insoluble flagellum/basal body fraction. However, we cannot rule out possible aggregation of the protein due to overexpression or the presence of the epitope tag. ARL2 is not a known component of the *T. brucei* flagellar/basal body proteome [38] but, like other GTPases, is likely to be found in very low abundance within the cell. In comparison to TbARL2, both human Arl2 and BART have been found associated with centrosomes throughout the cell cycle, so may be involved in the biogenesis of interphase microtubules and the mitotic spindle [50], but the majority of these proteins are located either in the cytosol or the mitochondria.

## 4 Discussion

As part of a broader study of ARFs/ARLs in kinetoplastids, we describe the characterization of an Arl2 orthologue in the lower eukaryote *T. brucei*. Arl2 has an essential role in the host bloodstream form of this parasite, with knockdown causing a defect in cleavage furrow formation and ingression. Cell cycle progression continues in the absence of cytokinesis, leading to the production of cells with multiple nuclei. Overproduction of TbARL2^NoTag^ has no effect on cell division but expression of TbARL2^myc^ results in incomplete furrow ingression. In comparison, the overexpression of GDP-locked Arl2 in HeLa cells causes cell cycle arrest in G2/M phase [50] but knockdown of Arl2 has no significant effect on cell division [50]. RNAi of the *Caernorhabditis elegans* Arl2 functional homologue CeEVL-20, however, causes severe defects in the microtubule cytoskeleton of postembryonically proliferating tissues and inhibits cytokinesis, leading to abnormal embryonic development [9]. Microtubule filament concentration is significantly decreased leading to effects on the cytokinetic cleavage furrow and cell cortex [9]. The *Schizosaccharomyces pombe* orthologue of Arl2, *alp41*(+), is essential for viability with disruption resulting in a complete loss of intact microtubules and growth polarity defects [8]. Similar effects are observed in *Saccharomyces cerevisiae* [51] and *Arabidopsis thaliana* [7]. Our data show that knockdown of Arl2 function in *T. brucei* results in loss of acetylated α-tubulin but not of total α-tubulin from cellular microtubules, with distinct inhibition of cleavage furrow formation and ingression but no obvious effects on the subpellicular microtubules.

The control of eukaryotic microtubule dynamics is of fundamental importance for correct maintenance of cell shape and polarity, progression through the cell cycle and intracellular trafficking. The central factor in microtubule biogenesis is the intrinsic property of α/β tubulin to polymerise in the presence of GTP but a plethora of effector and regulatory proteins are also involved in this process, including tubulin-specific chaperones, microtubule-associated proteins (MAPs), molecular motors and plus-end directed proteins, many of which have phosphorylation-state activation [52]. These regulatory mechanisms have particular significance in organisms with a tubulin-based cytoskeleton such as the kinetoplastids, which additionally require a tubulin-based axoneme for flagellar motility. Knockdown of α-tubulin itself in the *T. brucei* procyclic stage causes a defect in cell division and rounding up of parasites to produce the so-called “FAT” phenotype [53]. Knockdown of the same gene in BSF has proved problematic due to premature death of uninduced cell lines, which may be indicative of an extreme phenotype [54]. Arl2 and cofactor D make up two of the six components of the tubulin-cofactor system [55], of which only one other member, cofactor C, has previously been characterized in *T. brucei*. The trypanosome orthologue of cofactor C associates with α-tubulin at the transitional fibres originating from the mature basal body, destined for integration into the flagellar axoneme. RNA interference of cofactor C in procyclic stage *T. brucei* causes axonemal defects but has no effect on the subpellicular microtubules [49].

In the present study we provide the first evidence of a potential link between α-tubulin acetylation and Arl2 function. Acetylated α-tubulin is modified by the covalent attachment of an acetyl group to residue lysine 40 of the protein [56]. This form of modified tubulin has been associated with stable structures in eukaryotic cells, localizing to primary cilia, midbodies, centrioles and subsets of cytoplasmic microtubules in 3T3 and HeLa cells [57] and to flagella axonemes, basal bodies and cytoplasmic microtubules radiating from the basal bodies in *Chlamydomonas reinhardtii* [56,58]. Conversely, in *T. brucei*, acetylated tubulin is distributed widely throughout all microtubule arrays of the parasite, including the intranuclear mitotic spindle [48]. This post-translational modification appears to occur during or immediately after microtubule polymerisation, with the reverse process of deacetylation coinciding with depolymerisation [48,59,60]. The acetylation of α-tubulin does not have a significant effect on the rate of temperature-dependent polymerisation/depolymerisation *in vitro*, therefore this modification does not play a direct role in microtubule assembly [61]. Moreover, although polymerisation is not a requirement for this modification to occur, the tubulin polymer provides a better substrate for the reaction than tubulin heterodimers [61]. This correlates with biochemical analysis in *T. brucei* which found a lack of acetylated tubulin in the unpolymerised tubulin pool [48,62]. Although it was initially believed that acetylation was able to stabilise microtubules following construction, this hypothesis has since been challenged [63] and a mechanism by which this stabilisation may occur has not been elucidated. Rather, it has been suggested that microtubules may be stabilised by alternative means such as capping before acetylated tubulin begins to accumulate [63].

Despite the biochemical characterization of this post-translational modification in *Chlamydomonas* flagellar extracts over 20 years ago [61], the exact function of this modification is still largely unknown and enzymes with α-tubulin acetylating activity, N-acetyltransferase 10 (NAT10) and the neuronal Elongator, have only very recently been identified [64,65]. Interestingly the *T. brucei* orthologue of NAT10 is a component of the flagellar proteome [38]. The reverse process of deacetylation is catalysed by the histone deacetylase HDAC6 [66], which co-localizes with the microtubule end-tracking protein EB1 [67]. Overexpression of HDAC6 leads to total deacetylation of microtubules, whereas knockdown increases acetylation of tubulin in microtubules [66,67]. Knockdown of the *T. brucei* orthologue of HDAC6 is lethal but the effects of this enzyme on tubulin modification have not been published [68]. Further work is now required to determine the role of TbARL2 on α-tubulin acetylation and whether it directly influences either N-acetyltransferase or deacetylase function in the trypanosome.

## Appendix A Supplementary data

Supplementary Table 1 List of primer sequences.

Supplementary Table 2 Putative trypanosomatid orthologues of human ARL2-interacting proteins. aa, amino acids. Human sequences: Phosphodiesterase subunit delta (PDEδ/UNC119/HRG4), AF125998, 240 aa. Tubulin-folding cofactor D (cofactor D) AAH03094, 1192 aa. Protein phosphatase 2A regulatory subunit A alpha isoform (PP2A subunit Aα), P30153, 589 aa. Protein phosphatase 2A regulatory subunit B56 epsilon isoform (PP2A subunit B, B′, ɛ), NP006237, 467 aa. Protein phosphatase 2A regulatory subunit B alpha isoform (PP2A subunit B, B, α), AAV38550, 447 aa. Protein phosphatase 2A catalytic subunit C, beta isoform (PP2A subunit C, β), NP004147, 309 aa. Protein phosphatase methylesterase I (PMEI), NP057231, 386 aa. Binder of Arl Two (BART), NP036238, 163 aa. Adenine nucleotide translocator 1 (ANT), P12235, 298 aa. ELMOD domain containing protein 2 (ELMOD2), NP_714913, 293 aa. *BLAST searches using the human BART sequence identified no matches in *T. cruzi*. However, a further search using the putative *L. major* BART sequence revealed a putative *T. cruzi* orthologue of this protein (Tc00.1047053511279.30, 1.8e-32, 158 aa). The *T. brucei* sequence Tb10.05.0095 had the highest score for BLAST searches using both human and *L. major* putative BART orthologues.

Supplementary Fig. 1 Alignment of kinetoplastid ARL2 orthologues and related protein sequences. The conserved Switch I and II effector domains are shown in boxes. In agreement with other Arl2 orthologues, the TbARL2 protein sequence is predicted to form an amphipathic α-helix at the N-terminus and does not contain a consensus *N*-myristoylation motif. Sequence accession numbers (Genbank/GeneDB): Trypanosoma brucei, Tb10.70.3000. Trypanosoma cruzi, Tc00.1047053506295.80. *Leishmania major*, LmjF35.0130. Human, AAP35320.1. *Drosophila melanogaster*, Q06849. *Caenorhabditis elegans*, Q19705. *Plasmodium falciparum*, AAN37012. *Saccharomyces cerevisiae* (Yeast), P39110.

Supplementary Fig. 2 Alignment of putative kinetoplastid BART orthologues with related protein sequences. The human BART structure is composed of six helices [40,41] (marked here as boxed regions H1–H6). Corresponding helices have also been predicted for the *L. major* and *T. brucei* putative BART orthologues (also marked here in boxes). Sequence accession numbers (Genbank/GeneDB): Human, NP_036238.1. Mouse, EDL11139.1. *Xenopus laevis*, AAH74487.1. *Tetraodon nigroviridis*, CAF98910.1. *Aedes aegypti*, XP_001655639.1. *Trypanosoma cruzi*, Tc00.1047053511279.30. *Trypanosoma brucei*, Tb10.05.0095. *Leishmania major*, LmjF18.0120.

Supplementary Fig. 3 (A and B) Quantitative (Real Time) PCR to measure TbARL2-specific RNA transcript relative to *N*-myristoyltransferase (NMT) control in the Lister 427 parental cell line (wt) and the transfected cell lines 427/p2T7ARL2 (A), 427/pARL2^MYC^ and 427/pARL2NoTag (B), grown in the presence of tetracycline for 0–24 h. (C) Total lysates of cells grown in the absence or presence of tetracycline for 16 h were immunoblotted (1 × 10^7^ cells/lane) and probed with anti-myc or anti-EF-1α to monitor equal sample loading. 1, Lister 427 parental line; 2, 427/pARL2^MYC^ uninduced; 3, 427/pARL2^MYC^ + Tet; 4, 427/pARL2-T31N^MYC^ (GDP-locked) uninduced; 5, 427/pARL2-T31N^MYC^ + Tet; 6, 427/pARL2-Q70L^MYC^ (GTP-locked) uninduced; 7, 427/pARL2-Q70L^MYC^ + Tet. (D, E) Nucleus:kinetoplast configurations in the Lister 427 parental cell line (wt) and the transfected cell lines 427/p2T7ARL2 (D) and 427/pARL2^MYC^ (E), grown in the presence of tetracycline for 0–24 h. Any configurations other than 1K1N, 2K1N and 2K2N were classified as abnormal (MKXN). (F) Cumulative growth of the BSF transfected lines 427/pARL2-T31N^MYC^ (GDP-locked) and 427/pARL2-Q70L^MYC^ (GTP-locked) in the absence and presence of tetracycline over a 3-day time course. (G) Flow cytometry of propidium iodide stained cell lines 427/pARL2-T31N^MYC^ and 427/pARL2-Q70L^MYC^ grown in the presence of tetracycline for 0–24 h. DNA content of each peak is shown.

Supplementary Fig. 4 Transmission electron micrographs of cell line 427/p2T7ARL2 (RNAi) grown in the presence of tetracycline for 24 h. F, flagellum. FP, flagellar pocket. iF, internal flagellum. PMT, subpellicular microtubules. N, nucleus. K, kinetoplast. BB, basal body. FAZ PMT, quartet of subpellicular microtubules of the flagellum attachment zone. G, Golgi apparatus. c, cis-Golgi. t, trans-Golgi. CCV, clathrin-coated vesicle. Bar, 1 μm (A–C) or 200 nm (D).
