# Supplementary material for: The small GTPase ARL2 is required for cytokinesis in Trypanosoma brucei
Source: Mol Biochem Parasitol. 2010 Oct;173(2):123–31. doi: 10.1016/j.molbiopara.2010.05.016 (PMC2913242; doi:10.1016/j.molbiopara.2010.05.016)
Supplement: Supplementary Table 1 — List of primer sequences. [file mmc1.doc]

| **Primer** | **Sequence** |
| --- | --- |
| A2-RNAi-F | 5’-TCTTAGGTCTAGAGCGAGGTAGTATGGGT-3’ |
| A2-RNAi-R | 5’-CATATTAACCCATCAGTGCTCTAGAAGTAATTT-3’ |
| A2-myc-F | 5’-GGTGTAGTGCAAGCTTGTATGGGTTTAC-3’ |
| A2-myc-R | 5’-GTATCCAGTAGAATGTATTTTGCGGGGTCTAGATCCACAAATACTTGATGCAGGTATAAG-3’ |
| A2-T31N-F | 5’-GATTAGACAACGCAGGGAAGAACACGTGTGTGA-3’ |
| A2-T31N-R | 5’-TCTTCCCTGCGTTGTCTAATCCAAGCATCAG-3’ |
| A2-Q70L-F | 5’-ACATATGGGATGTTGGTGGTCTGCAGTCACTCCG-3’ |
| A2-Q70L-R | 5’-GACCACCAACATCCCATATGTTCAATGTGCA-3’ |
| A2-Untagged-F | 5’-GTAGAATGTATTTTGCGGGGTAGAGAGAACAGAA-3’ |
| A2-Untagged-R | 5’-ACCCCGCAAAATACATTCTACTGGATACATC-3’ |
| A2-qPCR-F | 5’-ACGTTGCTCGTTTGGATGACT-3’ |
| A2-qPCR-R | 5’-AGCCGTTCTTCCTGTAATAGTGTGT-3’ |
| α-Tub-qPCR-F | 5’-ATGCGTGAGGCTATCTGCAT-3’ |
| α-Tub-qPCR-R | 5’-CTAGTACTCCTCCACATCCTCCTCA-3’ |
| NMT-qPCR-F | 5’-CGAAAGCATGTTTCGATTCAAC-3’ |
| NMT-qPCR-R | 5’-GGTGATATCCCGGAGGCATA-3’ |

**Supplementary Table 1**
