# Supplementary material for: The small GTPase ARL2 is required for cytokinesis in Trypanosoma brucei
Source: Mol Biochem Parasitol. 2010 Oct;173(2):123–31. doi: 10.1016/j.molbiopara.2010.05.016 (PMC2913242; doi:10.1016/j.molbiopara.2010.05.016)
Supplement: Supplementary Table 2 — Putative trypanosomatid orthologues of human ARL2-interacting proteins. aa, amino acids. Human sequences: Phosphodiesterase subunit delta (PDEδ/UNC119/HRG4), AF125998, 240 aa. Tubulin-folding cofactor D (cofactor D) AAH03094, 1192 aa. Protein phosphatase 2A regulatory subunit A alpha isoform (PP2A subunit Aα), P30153, 589 aa. Protein phosphatase 2A regulatory subunit B56 epsilon isoform (PP2A subunit B, B′, ɛ), NP006237, 467 aa. Protein phosphatase 2A regulatory subunit B alpha isoform (PP2A subunit B, B, α), AAV38550, 447 aa. Protein phosphatase 2A catalytic subunit C, beta isoform (PP2A subunit C, β), NP004147, 309 aa. Protein phosphatase methylesterase I (PMEI), NP057231, 386 aa. Binder of Arl Two (BART), NP036238, 163 aa. Adenine nucleotide translocator 1 (ANT), P12235, 298 aa. ELMOD domain containing protein 2 (ELMOD2), NP_714913, 293 aa. *BLAST searches using the human BART sequence identified no matches in T. cruzi. However, a further search using the putative L. major BART sequence revealed a putative T. cruzi orthologue of this protein (Tc00.1047053511279.30, 1.8e-32, 158 aa). The T. brucei sequence Tb10.05.0095 had the highest score for BLAST searches using both human and L. major putative BART orthologues. [file mmc2.doc]

| **Protein** | ***T. brucei* orthologue** | **E Value** | **AA** | ***T. cruzi* orthologue** | **E value** | **AA** | ***L. major* orthologue** | **E value** | **AA** |
| --- | --- | --- | --- | --- | --- | --- | --- | --- | --- |
| PDEδ | Tb927.2.4580 | 2.5e-38 | 196 | Tc00.1047053506979.50 | 9.3e-43 | 196 | LmjF27.2000 | 5.2e-41 | 197 |
| Cofactor D | Tb927.8.6200 | 9.8e-118 | 1343 | Tc00.1047053511067.10 | 5.5e-129 | 1314 | LmjF24.2020 | 1.6e-115 | 1445 |
| PP2A subunit Aα | Tb927.1.1380 | 2.7e-80 | 573 | Tc00.1047053506479.39 | 2.2e-82 | 573 | LmjF20.0660 | 2.1e-89 | 599 |
| PP2A subunit B, B’, ε | No match | - | - | No match | - | - | No match | - | - |
| PP2A subunit B, B, α | Tb11.01.6480 | 7.1e-28 | 732 | Tc00.1047053506221.100 | 4.6e-30 | 732 | LmjF33.0670 | 5.6e-28 | 876 |
| PP2A subunit C, β | Tb927.3.1240 | 3.6e-126 | 323 | Tc00.1047053509453.50 | 2.3e-126 | 340 | LmjF25.1320 | 9.4e-129 | 317 |
| PMEI | Tb11.01.5750 | 7.1e-33 | 328 | Tc00.1047053511727.100 | 6.8e-32 | 331 | LmjF32.0910 | 1.0e-37 | 280 |
| BART | Tb10.05.0095 | 0.10 | 127 | *Tc00.1047053511279 | * | 158 | LmjF18.0120 | 1.9e-06 | 127 |
| ANT | Tb10.61.1820 | 1.2e-77 | 307 | Tc00.1047053511289.70 | 6.3e-76 | 314 | LmjF19.0200 | 9.1e-76 | 317 |
| ELMOD2 (a) | Tb09.160.1330 | 2.6e-10 | 369 | Tc00.1047053508347.70 | 6.7e-09 | 375 | LmjF19.1570 | 3.7e-13 | 248 |
| ELMOD2 (b) | Tb10.70.0940 | 1.0e-08 | 459 | Tc00.1047053506327.70 | 6.9e-07 | 477 | LmjF26.1850 | 4.9e-11 | 418 |

**Supplementary Table 2**
