# Supplementary material for: The small GTPase ARL2 is required for cytokinesis in Trypanosoma brucei
Source: Mol Biochem Parasitol. 2010 Oct;173(2):123–31. doi: 10.1016/j.molbiopara.2010.05.016 (PMC2913242; doi:10.1016/j.molbiopara.2010.05.016)
Supplement: Supplementary Fig. 1 — Alignment of kinetoplastid ARL2 orthologues and related protein sequences. The conserved Switch I and II effector domains are shown in boxes. In agreement with other Arl2 orthologues, the TbARL2 protein sequence is predicted to form an amphipathic α-helix at the N-terminus and does not contain a consensus N-myristoylation motif. Sequence accession numbers (Genbank/GeneDB): Trypanosoma brucei, Tb10.70.3000. Trypanosoma cruzi, Tc00.1047053506295.80. Leishmania major, LmjF35.0130. Human, AAP35320.1. Drosophila melanogaster, Q06849. Caenorhabditis elegans, Q19705. Plasmodium falciparum, AAN37012. Saccharomyces cerevisiae (Yeast), P39110. [file mmc3.pdf]

```
                                                                        Switch I
Tbrucei      MGLLSIIRKTKRKEREMRILMLGLDNAGKTTCVKKFCGKD---TSSISPTLGFQITAFSLNGCTL 62
Tcruzi       MGLLSIIRKTKRKEREMRILMLGLDNAGKTTCIKKFCGKD---TSSISPTLGFQITAFSLHGCTL 62
Lmajor       MGLLSIIKKTKRKEREMRILMLGLDNAGKTTCVKKLCGKD---TSLISPTLGFQITALTFRGCTL 62
Human        MGLLTILKKMKQKERELRLLMLGLDNAGKTTILKKFNGED---IDTISPTLGFNIKTLEHRGFKL 62
Drosophila   MGFLTVLKKMRQKEREMRILLLGLDNAGKTTILKRFNGEP---IDTISPTLGFNIKTLEHNGYTL 62
Celegans     MGFLKILRKQRAREREMRILILGLDNAGKTTLMKKFLDEP---TDTIEPTLGFDIKTVHFKDFQL 62
Plasmodium   MVLLKILKKIKDNKRNLRILILGLDNAGKTTIIKRLLGED---IYSVSPTFGFNIETLEFGNNIL 62
Yeast        MGLLSIIRKQKLRDKEIRCLILGLDNSGKSTIVNKLLPKDEQNNDGIMPTVGFQIHSLMIKDVTI 65
             * :*.:::* : ..:::* *:*****:**:* ::::  :       : **.**:* :.   .  :

                Switch II
Tbrucei      NIWDVGGQQSLRSYWRNYFESTDGLIWVVDSNDVARLDDCRRELHTLLQEER---LAGASLLILL 124
Tcruzi       NIWDVGGQQSLRSYWRNYFESTDGLIWVVDSNDLARLEDCRKELHALLQEER---LAGASLLVFL 124
Lmajor       NVWDVGGQQSLRSYWRNYFESTDGLVWVVDSNDVERLLMCKEELHHLLQEER---LAGASLLVFL 124
Human        NIWDVGGQKSLRSYWRNYFESTDGLIWVVDSADRQRMQDCQRELQSLLVEER---LAGATLLIFA 124
Drosophila   NMWDVGGQKSLRSYWRNYFESTDGLVWVVDSADRMRLESCGQELQVLLQEER---LAGATLLVLC 124
Celegans     NLWDVGGQKSLRSYWKNYFESTDALIWVVDSSDRERLLQCSEELKKLLGEER---LAGASLLVLA 124
Plasmodium   NIWDIGGQKSIRHFWKNYYEDVDGIIFVVDSTDLFRLQLCSFELKQILKEER---LYGSTLLILS 124
Yeast        SLWDIGGQRTLRPFWDNYFDKTQAMIWCIDVSLSMRFDETLQELKELINRDENRIGYECAVIVVL 130
             .:**:***:::* :* **::..:.::: :*     *:     **: :: .:.       .::::.

Tbrucei      NKQDLS---GALPPGEIERHLGIDIIRKGKRHVYLCACSAKTGAGLLQGMEWIVQDVSSRMYFAG 186
Tcruzi       NKQDLP---AAISPVEIAQHLDIDTIKKGKRHVQLCACSAKTGDGLLEGMEWIVRDVSSRMYFAS 186
Lmajor       NKIDIP---TALSPQEIAGLLDVDTIRQGKRHVHLCACSARTGEGLLDGISWMVDDVSKRMYFSS 186
Human        NKQDLP---GALSSNAIREVLELDSIRS--HHWCIQGCSAVTGENLLPGIDWLLDDISSRIFTAD 184
Drosophila   NKQDLP---GALSSNEIKEILHLEDITT--HHWLVAGVSAVTGEKLLSSMDWLIADIAKRIFTLD 184
Celegans     NKSDLP---GAIDVNSIAQVLDLHSIKS--HHWKIFSCCALSGDRLVQAMTWLCDDVGSRLFILD 184
Plasmodium   NKVDID---KSLTINQIVEILKLNEMNMD-RHWCINECSAFSGKGLLKSFMWLIDDITDRIDS-- 183
Yeast        NKIDLVEDKSELHRRCLLVESELKCLFKPDIRIELVKCSGVTGEGIDNLRDRLVESCHFTQ---- 191
             ** *:      :    :     :. :     :  :   .. :*  :      :  .
```

Supplementary Figure 1
