# Supplementary figures and images for: The small GTPase ARL2 is required for cytokinesis in Trypanosoma brucei
Source: Mol Biochem Parasitol. 2010 Oct;173(2):123–31. doi: 10.1016/j.molbiopara.2010.05.016 (PMC2913242; doi:10.1016/j.molbiopara.2010.05.016)

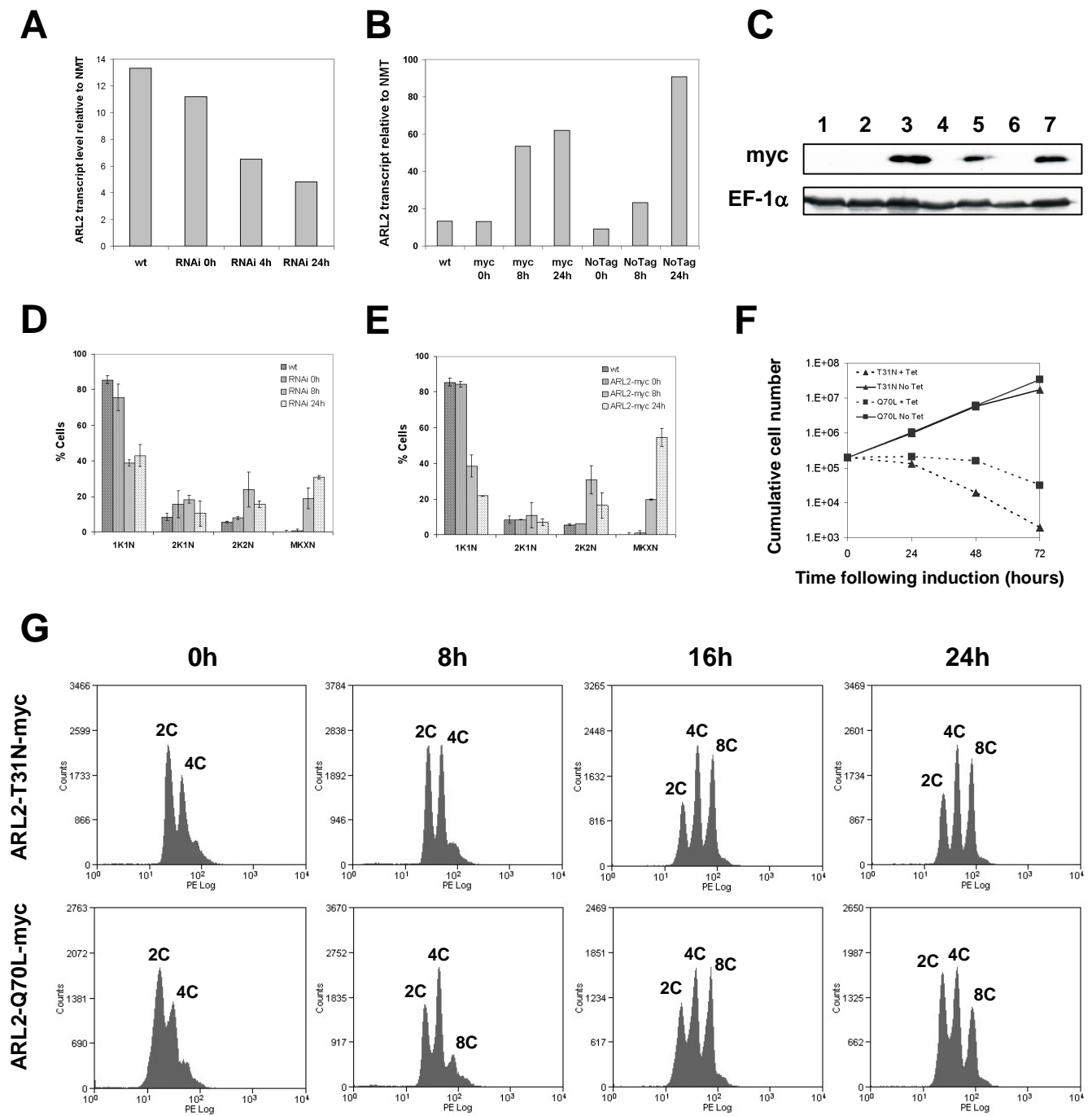

Supplementary Figure 3

Supplement: Supplementary Fig. 3 — (A and B) Quantitative (Real Time) PCR to measure TbARL2-specific RNA transcript relative to N-myristoyltransferase (NMT) control in the Lister 427 parental cell line (wt) and the transfected cell lines 427/p2T7ARL2 (A), 427/pARL2MYC and 427/pARL2NoTag (B), grown in the presence of tetracycline for 0–24 h. (C) Total lysates of cells grown in the absence or presence of tetracycline for 16 h were immunoblotted (1 × 10^7^ cells/lane) and probed with anti-myc or anti-EF-1α to monitor equal sample loading. 1, Lister 427 parental line; 2, 427/pARL2MYC uninduced; 3, 427/pARL2MYC + Tet; 4, 427/pARL2-T31NMYC (GDP-locked) uninduced; 5, 427/pARL2-T31NMYC + Tet; 6, 427/pARL2-Q70LMYC (GTP-locked) uninduced; 7, 427/pARL2-Q70LMYC + Tet. (D, E) Nucleus:kinetoplast configurations in the Lister 427 parental cell line (wt) and the transfected cell lines 427/p2T7ARL2 (D) and 427/pARL2MYC (E), grown in the presence of tetracycline for 0–24 h. Any configurations other than 1K1N, 2K1N and 2K2N were classified as abnormal (MKXN). (F) Cumulative growth of the BSF transfected lines 427/pARL2-T31NMYC (GDP-locked) and 427/pARL2-Q70LMYC (GTP-locked) in the absence and presence of tetracycline over a 3-day time course. (G) Flow cytometry of propidium iodide stained cell lines 427/pARL2-T31NMYC and 427/pARL2-Q70LMYC grown in the presence of tetracycline for 0–24 h. DNA content of each peak is shown. [file mmc5.pdf]

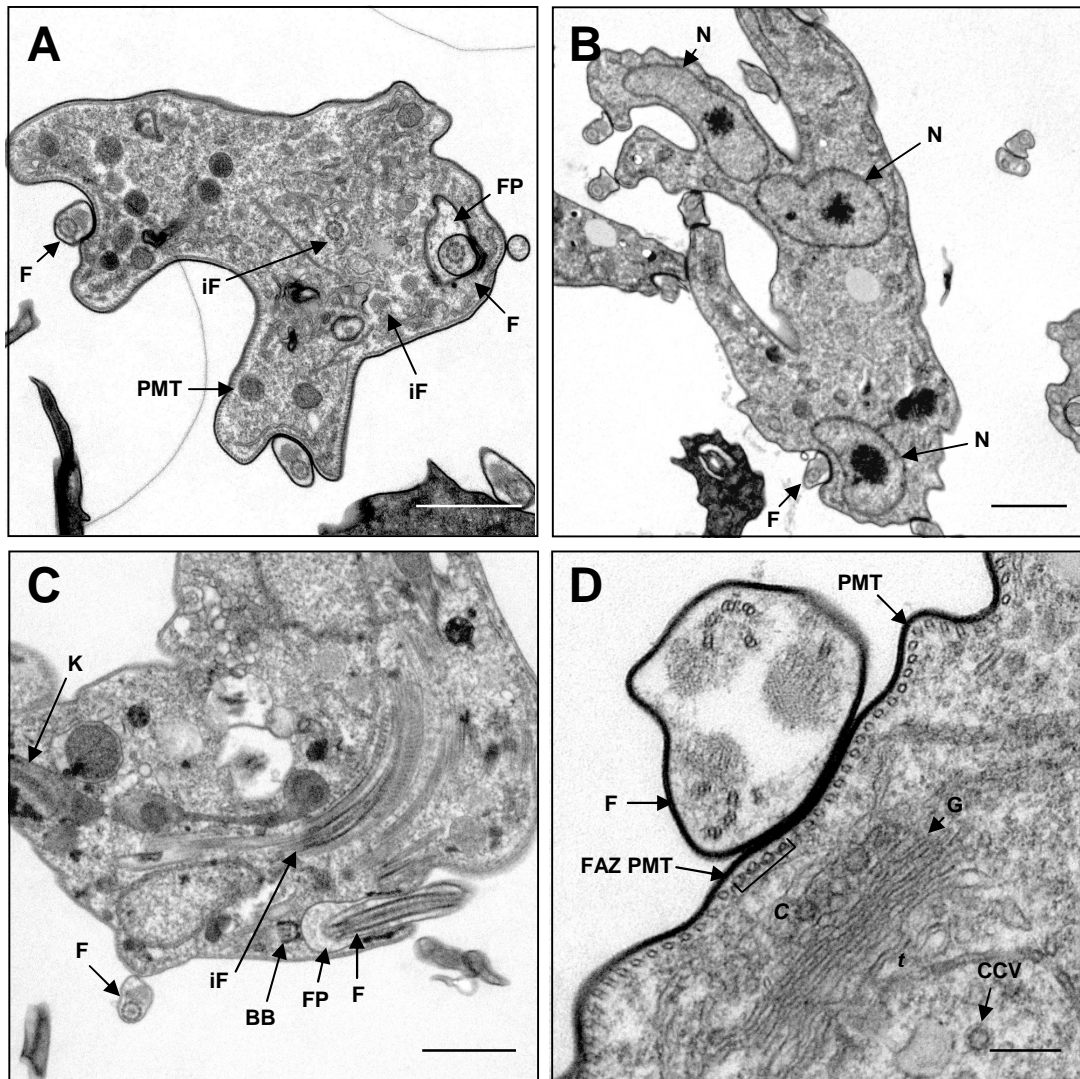

**Supplementary Figure 4**

Supplement: Supplementary Fig. 4 — Transmission electron micrographs of cell line 427/p2T7ARL2 (RNAi) grown in the presence of tetracycline for 24 h. F, flagellum. FP, flagellar pocket. iF, internal flagellum. PMT, subpellicular microtubules. N, nucleus. K, kinetoplast. BB, basal body. FAZ PMT, quartet of subpellicular microtubules of the flagellum attachment zone. G, Golgi apparatus. c, cis-Golgi. t, trans-Golgi. CCV, clathrin-coated vesicle. Bar, 1 μm (A–C) or 200 nm (D). [file mmc6.pdf]
