# Supplementary material for: The small GTPase ARL2 is required for cytokinesis in Trypanosoma brucei
Source: Mol Biochem Parasitol. 2010 Oct;173(2):123–31. doi: 10.1016/j.molbiopara.2010.05.016 (PMC2913242; doi:10.1016/j.molbiopara.2010.05.016)
Supplement: Supplementary Fig. 2 — Alignment of putative kinetoplastid BART orthologues with related protein sequences. The human BART structure is composed of six helices [40,41] (marked here as boxed regions H1–H6). Corresponding helices have also been predicted for the L. major and T. brucei putative BART orthologues (also marked here in boxes). Sequence accession numbers (Genbank/GeneDB): Human, NP_036238.1. Mouse, EDL11139.1. Xenopus laevis, AAH74487.1. Tetraodon nigroviridis, CAF98910.1. Aedes aegypti, XP_001655639.1. Trypanosoma cruzi, Tc00.1047053511279.30. Trypanosoma brucei, Tb10.05.0095. Leishmania major, LmjF18.0120. [file mmc4.pdf]

```
                                                                         H1
HUMAN         ------------------------------MDALEGESFALSFSSAS--DAEFDAVVGYLEDIIMD 34
MOUSE         -----------------------------------------MRSSAS--DAEFDAVVGCLEDIIMD 23
XENOPUS       ------------------------------MEDLEEENFSLSVSSPK--DAEFDNVVGHLEDIIMD 34
TETRAODON     -------------MDVQEQNGRSCGENIVDMVDLEDENLASSSSSAT--DASFDAVIGCIEDIIME 51
AEDES         -------------------------------MDFEEN---LVKRQNS--TEYFDTVIGHIEDIVIG 30
TCRUZI        MWCSFIFIRWSCFLFFFFFFFFFGAFPGKKEMDTEDGEFMVCGSGGTVEDAQLDDLVGVIEDFMAH 66
TBRUCEI       -------------------------------MDSEEGEFVVYGDCGSAEDAQFDQLVGAIEDFMVN 35
LMAJOR        -------------------------------MDTEEGEFIICGNGGSPEDAAFDAVVGVIEDFMIS 35
                                                            .     :* ::* :**::

                      H2                              H3                   H4
HUMAN         DEFQLLQRNFMDKYYLEFEDTEENKLIYTPIFNEYISLVEKYIEEQLLQRIPEFN-MAAFTTTLQH 99
MOUSE         DEFQLLQRNFMDKYYQEFEDTEENKLTYTPIFNEYISLVEKYIEEQLLERIPGFN-MAAFTTTLQH 88
XENOPUS       DEFQLLQHGFMDKHYHEFEDTEENKLTYTTIFNEYIGLVEKYIEEQLLQRIPAFD-MSAFTSSLQC 99
TETRAODON     EKFQQLQQSFMEKHYLEFDDSDENKLSYTTIFNEYVSLLEKHLEQQLMQRIPGFN-MNTFTEVLMQ 116
AEDES         EDFQTMVNHFMDCYYHLFDQGEENKIVYTEIYQKYTSLIETFIVESLNAKMSYFD-MDRFAVELEN 95
TCRUZI        FDPDAVFRQLPPFSSVASDHERHR------LYREAVLQTEADLDAYVLEHCGSIESLKDATSLILD 126
TBRUCEI       LDQDAMLAKLPPFFSVSDEHERHK------IHRELLKRVDADLDEHVLKNCQSIGSMENAVRILES 95
LMAJOR        FDPEQVWQSLPPLHTVSGDHDQHT------VYTSFLEKVDQELDAHVLASCPEYKSIEQVVTLLQK 95
               . : :   :        :.  .       :. .     :  :   :         :   .  :

                         H5             H6
HUMAN         HKDEVAGDIFDMLLTFTDFLAFKEMFLDYRAEKEGRGLDLSSGLVVTSLCKSSSLPASQNNLRH 163
MOUSE         HKDEVAGDIFDMLLTFTDFLAFKEMFLDYRAEKEGRGLDLSSGLVVTSLCKSSSTPASQNNLRH 152
XENOPUS       HREEIAGDIFDILLTFTDFLAFKEMFLDYKAEKEGRTVDLGCGLVVTSLM-SSSISSS------ 156
TETRAODON     HKEEVSGDIFDMLLTFTDFLAFKEMFLAYRTEKEGRGLDLSQGLLITPLV-------------- 166
AEDES         KKSQLDGEIFELLYTLTDFLAFKDMVLDYKAFKEGVYDDLSKDFSVTSLQQRQ----------- 148
TCRUZI        RSDEIAEEVRDFIN--EGCFNYTTFVELWKQNKK------------------------------ 158
TBRUCEI       RKEEISEDVLDFVS--DGFLDYNIFVEAWEKRDQ------------------------------ 127
LMAJOR        RHEDITEEVWSFVS--EGCFDYEAFMEQWKKKRP------------------------------ 127
              : .::  :: .::    . : :  :.  :.
```

Supplementary Figure 2
